# Correlational study between online friendship network and internet game disorder among university students

**DOI:** 10.1002/brb3.2392

**Published:** 2021-10-17

**Authors:** Sung‐Min Son, Jin‐Seok Oh, Byoung‐Jin Jeon

**Affiliations:** ^1^ Department of Occupational Therapy Jeonju Kijeon College Jeonju South Korea; ^2^ Department of Emergency Medical Rehabilitation Service Graduate School of Kangwon National University Samcheok South Korea; ^3^ Department of Occupational Therapy Kangwon National University Samcheok South Korea

**Keywords:** correlation analysis, internet game disorder, online friendship, social network analysis, university students

## Abstract

**Purpose:**

The purpose of this study was to provide basic information about the analysis of the correlation between online friendship network and internet game disorder among university students.

**Methods:**

Study subjects were 77 university students. For analysis of online friendship among them, social network analysis was performed and the analysis of degree, closeness, and betweenness centrality was measured. Assessment of internet game disorder was done using the Korean version of Internet Gaming Disorder Scale.

**Results:**

As per the results, the positive correlation showed between in‐closeness centrality and internet gaming disorder (IGD) and the negative correlation showed between out‐closeness centrality and internet gaming disorder.

**Conclusions:**

Online friendship is considered to contribute to the changes of internet game disorder level. Thus, in the relationship between online friendship and internet game disorder, closeness centrality should be considered, and causal relation analysis should be performed in further studies.

## Graphical abstract

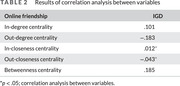

## 1 INTRODUCTION

The period of time students spend in a university is crucial for them to develop maturity and is reported to be the time in which students learn the responsibilities, norms, and roles that will be required of them as adults (Lee et al., 2007). University students not only decide their careers during this period, but also acquire social status and positions through their occupations and perform social roles that enhance their sense of self‐worth and fulfill their ego (Seo & Seong, 2020). The most important psychosocial development task for university students throughout this process is to escape adolescence, a phase in which they used to form social relationships within a restricted environment. During university, young adults begin experiencing mature interpersonal dynamics through which they expand their social relationships and learn how to properly form interpersonal relationships (Lee et al., 2007). Over time, the focus of development during this period becomes centered on cultivating social skills that enable students to form close interpersonal relationships with others (Hong & Jeon, 2017). Thus, university students seek to directly form the various interpersonal relationships in offline zones, such as university societies and the corresponding activities, utilizing social network services (SNS) such as KakaoTalk, Facebook, and Instagram to freely and dynamically form interpersonal relationships online (Kwon et al., 2013).

According to the trend of the fourth Industrial Revolution, the use of smartphones is increasing, online communication is becoming more common, and online interpersonal relationships among university students are continuously growing, making the use of connective technological devices an inevitable part of university students’ lives (Lee et al., 2011). The online space reduces the intensity of sensory stimuli that are generated in offline spaces, thus enabling inner emotions to be more easily expressed. This promotes the formation of new relationships and has an advantage of allowing individuals to maintain relationships with close friends in offline spaces (Leung, 2002). Such factors have increased online communications and have demonstrated a favorable function of promoting relationships with close friends in offline spaces (Kwon et al., 2013). However, online interpersonal relationships also have an adverse function of only strengthening superficial relationships with strangers (Leung, 2002). Such relationships have been reported to interfere with or limit the friendship and reduce the amount of time for exchange in the actual offline interactions between friends. Moreover, online relationships have been reported to have a trait of possibly leading to unbeneficial relationships (Kwon et al., 2013).

Both the positive and adverse effects of online interpersonal relationships can have different effects on an individual according to who they establish the relationship with (Kwon et al., 2013). In general, increased communication in close relationships promote self‐expression, which leads to a positive effect in terms of maintaining close relationships. However, when the purpose of the relationship is toxic, the online activities, the perceived depth of connection, and the details of the activities may change negatively depending on how often the individual interacts with and contacts the other party who has a toxic purpose (Hsu et al., 2011). In terms of analyzing interpersonal relationships, the theory of social network analysis (SNA), structurally studies the characteristics of relationships by reflecting actual interactions from the perspective of groups and individuals. This theory suggests that a higher number of online relationships with others, and being in closer proximity within the groups of relationships by structure, can lead to greater impacts and repercussions from the relationship, which increases the likelihood of sharing the negative purpose of the relationship. Furthermore, transfer and exchange of information is actively promoted in this process, leading to an increase and absorption of negative behaviors that are reported to cause addiction, disability, and overdependence (Jung & Baek, 2008).

From the various problems that can arise from the negative effects of online interpersonal relationships induced by the increase in communication due to the use of SNS, the majority of previous studies have referred to the internet gaming disorder (IGD) (Kim & Kang, 2012; Kim, 2013). According to the revised International Classification of Diseases (ICD), IGD is when an individual loses control over gaming, considers gaming more essential than other daily activities, and continues to play games for more than 12 months despite detrimental consequences. Furthermore, it is classified as a mental disorder in which the urge cannot be controlled by the individual, such as gambling addiction (WHO, 2018). IGD is also medically classified as an impulse control disorder, and the increasing overuse of gaming is known to cause maladjustment problems in daily life, as well as other issues that arise in various forms such as dependence on games (Choi & Son, 2011). IGD is largely caused by stress, with the major causes of IGD for university students being the stress that occurs from the changes in lifestyle, time management, and adaptation into adulthood that they did not experience in adolescence, the burden of study and employment, and the uncertainties of the future that they experience during this transition (Kim, 2013).

The main symptoms of IGD are insomnia, headache, sleep anxiety (Kwon et al., 2005), deteriorating symptoms of ADHD (Kwon et al., 2013), study negligence and decline in achievement levels (Gentile et al., 2004), obsessive‐compulsive disorder, attention deficit disorder (Chan & Rabinowitz, 2006; Nam & Lee, 2005), dissociative disorders, and increased aggression (Grusser et al., 2005). IGD is also known to lead to running away from home and suicidal tendencies when the condition becomes severe (Gyeong et al., 2012). According to the 2019 survey on smartphone dependence by the National Information Society Agency (NIA), IGD is more prominent in university students than adolescents, and the incidence rate is consistently increasing every year (NIA, 2019). A study by Ryu and Kim (2018) reported that university students use 57% of their total smartphone use hours throughout the day on internet gaming and that future studies must acknowledge university students as a group vulnerable to IGDs as they had the highest internet gaming time among all other age groups.

In addition, their study outlined that online communication could encourage students to share information on online games and increase the level of exchange between students because various SNS platforms that can promote online communication are widespread in the online environment. Furthermore, online interpersonal relationships formed through SNS have been reported to encourage participation into online games (Kwon et al., 2005). This has increased interest and engagement in online games, which in turn has led to a higher rate of online gaming (Gentile et al., 2004). Consequently, various studies on the use of SNS targeting university students have been conducted, such as the studies by Choi et al. (2018) and Shin and Yim (2019). A number of other studies assessing online friend relationships formed on SNS have also been reported, including a study by Kwon et al. (2013). A plethora of studies have also evaluated IGDs targeting various age groups, such as the studies by Ko et al. (2014), Seo and Seong (2020), Shu et al. (2019), and Zhang et al. (2019).

Despite IGD having emerged as a social concern, there is still an insufficient number of studies that have analyzed online gaming disorders targeting university students, who are the most predominant users of online games. The majority of existing studies associated with online gaming disorders are limited to analyzing the degree and the negative effects of gaming such as the amount of time spent on online gaming and the degree of immersion. Consequently, there is a limit to explaining the behavior of use in individuals with IGD. In addition, no studies have researched the relationship between online gaming disorders and online interpersonal relationships as a factor of such disorders.

Based on these, this study was conducted to verify the hypothesis that there is a relationship between online friendship network and IGD among university students. Accordingly, the purpose of this study is to utilize the degree of social networks to reflect online interpersonal relationships into real interactions using SNA, and thereby analyze the correlation with IGD by structurally evaluating the characteristics of online interpersonal relationship and then this study will also provide the basic information about the correlation analysis.

## 2 METHODS

### 2.1 Subjects

The participants of this study were a total of 77 university students attending the Department of Occupational Therapy at University in Chungcheongbuk‐do. Based on the research by Hwang et al. (2012), convenience samples were extracted from those who did not have a diagnosis of intellectual disability or organic mental disorder at the time of research, those without a medical history, and those who consented to participate in this study. In addition, the exclusion criterion for this study outlined participants must not be taking antipsychotics, must not have audiovisual problems that may cause problems in the evaluation, and must not have neurological orthopedic problems in body structure function.

The selection of the number of participants was also based on a study by Hwang et al. (2012). The G*Power 3.1.9.4 program was used to calculate the number of participants required for correlation analysis in this study with an effect size of .50, a significance level of 50, a statistical power of .90, and a two‐sided test, in accordance with the purpose of this study. As a result, a minimum of 42 participants were required. Therefore, a total of 77 university students were selected in the final stage to improve reliability in the progress of the research and by considering the expected dropout rate of the participants.

Prior to receiving their consent, all participants were provided a full description of the purpose and method of this study for their understanding. Following this step, the participants voluntarily agreed to participate in this study. The consent to participate in the study was provided online in a written form in consideration of the social circumstances following the COVID‐19 pandemic. Based on the ethical principles of the Helsinki Declaration, this process was conducted.

This study was approved by the Kangwon National University Institutional Review Board (IRB approval No.: KWNUIRB‐2020‐02‐010‐002). The study was conducted from May 1, 2021 to May 30 of the same year, for a total duration of four weeks.

### 2.2 Study procedure

This study applied a single group study design and performed a correlation analysis to assess the correlation between online friendships and IGD for university students. Considering the social circumstances following the spread of COVID‐19, the evaluation of the participants' online friendships and IGD was conducted in the form of an online questionnaire. Prior to the evaluation, the purpose and the method of the evaluation were sufficiently explained. The participants were allowed to partake in the evaluation after confirming that they understood the explanation of the study. In addition, the evaluation was conducted in a comfortable and stable condition, and participants provided individual responses to the evaluation. The evaluation was conducted by two occupational therapist who were the researchers of this study: One of them personally took care of the investigation and the other scored the tests.

### 2.3 Analysis of online friendship

The analysis was conducted using SNA, which enables an analysis of the characteristics of relationships formed through real‐life interactions. The SNA was conducted by specifically separating the data collection and analysis. The data was collected through a structured questionnaire on online friendships, with open‐ended questions such as writing the names of the participants’ best online friends. Participants were asked to write up to three names of their best online friends and were provided with "blank" or "none" options if they could not fill the spaces. Considering the characteristic of SNA, which analyzes groups with structural range restrictions, the names of friends from other universities not attended by the participant were excluded in the analysis. Lastly, based on a study by Marden (2005), the intensity was reflected through the number of meetings, which had the significance of forming a close relationship over a certain period of time.

To analyze the online friendships, centrality analysis was conducted using the SNA. The degree centrality, closeness centrality, and betweenness centrality were analyzed as the indicators for the analysis.

### 2.4 Internet game disorder

To evaluate the IGD of the participants, this study used the Korean version of IGD scale (K‐IGDS), which was adapted and validated into Korean (Cho & Kwon, 2017). The K‐IGDS was formed from the Internet Gaming Disorder Scale (IGDS), which was developed and validated according to the diagnostic criteria of DSM‐V in a study by Lemmens et al. (2015). According to the diagnostic criteria of DSM‐V, K‐IGDS is a tool with high efficacy that can sensitively measure the degree of IGD. K‐IGDS consists of 9 detailed factors and has a total of 27 questions with 3 questions for each factor. Each factor is evaluated on a 5‐point Likert scale based on the experience over the past year. The 0 point signify "never," 1 point signifies “1−4 times a year,” 2 points signify “5−11 times a year,” 3 points signify “1−3 times a month,” 4 points signify “once a week,” and 5 points signify “every day or almost every day.” The total score of K‐IGDS ranges from 0 to 155, with higher scores indicating higher severity of IGD. The Cronbach's *α* value for the reliability of this K‐IGDS tool was reported as high at .96. The reliability of K‐IGDS analyzed in this study was also high at .84.

### 2.5 Statistical analysis

The collected data for each question was encoded and analyzed using SPSS version 23.0. The general characteristics of the participants were analyzed using descriptive statistics, and the correlation between online friendships and IGD in university students was analyzed using correlation analysis. In addition, SNA was used to analyze online friendships through the Netminer 4.0 software. The statistical confidence level of this study was set to 95%.

## 3 RESULTS

### 3.1 General characteristics of subjects

Descriptive statistics was used to analyze the characteristics of study subjects. The results of characteristics of study subjects are shown in Table 1. Study subjects were 77 university students. In average, male showed 21.94 years and female showed 20.46 years. In sex, it was divided into 53 males and 24 females. In terms of university years, they showed 2 years (*n* = 49) and 3 years (*n* = 28). In game frequency, they played a game every day (*n* = 15), three or four times a week (*n* = 28), one or two times a week (*n* = 17), and not at all (*n* = 17). In terms of game time, they played a game below 1 h (*n* = 25), above 1 h to below 2 h (*n* = 15), above 2 h to below 3 h (*n* = 28), above 3 h to below 4 h (*n* = 5), and above 4 h (*n* = 4).

**TABLE 1:** Characteristics of study subjects (*n* = 77)

Characteristics	Items	Mean ± SD.
Age (years)	Male	21.94 ± 1.54
	Female	20.46 ± 0.83
Sex (*n*, %)	Male	53 (68.80)
	Female	24 (31.20)
University years (*n*, %)	2nd grade	49 (63.63)
	3nd grade	28 (36.36)
Game frequency (*n*, %)	Everyday	15 (19.50)
	3 or 4 times a week	28 (36.40)
	1 or 2 times a week	17 (22.10)
	Not at all	17 (22.10)
Game time (*n*, %)	Below 1 h	25 (32.50)
	Above 1 h to below 2 h	15 (19.50)
	Above 2 h to below 3 h	28 (36.40)
	Above 3 h to below 4 h	5 (6.50)
	Above 4 h	4 (5.20)

### 3.2 Results of correlation between online friendship and IGD

The results of correlation between online friendship and IGD are shown in Table 2. The positive correlation showed between in‐closeness centrality and IGD, and as the results of statistical verification of these, the statistically significant difference was shown at the 95% significant level. Thus, if the level of in‐closeness centrality showed an increase, the IGD showed an increase. The negative correlation showed between out‐closeness centrality and IGD, and as the results of statistical verification of these, the statistically significant difference was shown at the 95% significant level. Thus, if the level of out‐closeness centrality showed an increase, the IGD showed a decrease. However, in the other results of centrality, the in and out degree centrality and betweenness centrality did not show statistically significant difference with IGD.

**TABLE 2:** Results of correlation analysis between variables

Online friendship	**IGD**
In‐degree centrality	.101
Out‐degree centrality	−.183
In‐closeness centrality	.012*
Out‐closeness centrality	−.043*
Betweenness centrality	.185

* *p* < .05; correlation analysis between variables.

## 4 DISCUSSION

As a result of analyzing the correlation between online interpersonal relationships and IGD in this study, statistically significant differences were found in the in‐closeness centrality and out‐closeness centrality. A positive correlation was found for in‐closeness centrality as an increase in in‐closeness centrality showed an increase in the degree of IGD. The term link in SNA is described as a relationship formed based on preferential attachment rather than random connection between participants. In such a link, the direction of the relationship from another person to oneself is classified as “in,” and the direction of the relationship from oneself to another is classified as “out” (Albert & Barabasi, 2002). From the indicators used to determine the degree of the social network, closeness centrality is an indicator that measures the degree of a relationship based on the distance in the relationship between two individuals. It has a characteristic of reflecting all links that are directly or indirectly connected in the network, and is calculated by adding the distances of all the relationships (Cho & Bang, 2009). Therefore, closeness centrality is used to effectively explain the detailed concept of centrality in relationships as an analysis index of interpersonal relationships.

The result of analyzing SNA in a study by Cho and Bang (2009) described high in‐closeness centrality as being closely located to others within the network, which increases the likelihood of being located at the center of the network. Therefore, the direction of information becomes concentrated to one individual, the exchange of information is promoted, and the possibility of acquiring information increases dramatically. This reflects a structural characteristic of enabling the formation of new links or being affected by existing links. It can lead to being in close proximity with others in online interpersonal relationships, which promotes the sharing and exchange of information about the game and results in continuous exposure. This process is predicted to increase the use of online games. In addition, the level of immersion in online games also increases due to the influence of others who use the games, which can readily lead to IGD, causing problems of overdependence and maladjustment in daily lives.

A study by Kim and Park (2007) also reported that online games facilitate interactions between humans and computers, as well as direct interactions with other gamers. Moreover, it also enables 1:1 interactions or one user against multiple users and provides simultaneous accessibility to perform mutual interactions simultaneously in a virtual space with numerous other users. Such characteristics play a role as a factor that can induce the user to frequently contact others in the process of gaming and can formulate an online interpersonal relationship with another individual who plays the game. As a consequence, university students are placed in a position where they can be negatively influenced by gaming friends they meet in the online space. In addition, the degree of immersion in online games is promoted due to the increase in communication that occurs in the relationship, which can easily lead to IGD. A study by Cho et al. (2006) also outlined that online interpersonal relationships increase interaction in the online space and induce immersion in performing such actions. Furthermore, the study reported that the relationships significantly increase the game playing time, causing considerable problems in the daily lives of users. Once the user experiences an optimal state of immersion, it can lead to a level of addiction beyond the user's control.

The results of analyzing networks using media by Bae and Park (2007) also showed a higher probability of being in the center of the network with a higher degree of closeness centrality, which leads to attaining a position that receives recognition. Achieving a position that gains social support leads to sharing the information obtained through media, and enables the individual to make certain topics as a common or shared interest through the interactions that occur in the group. Such results are also consistent with the results of this study. Individuals with a high degree of closeness centrality are placed in close proximity with others in the network, which enables them to respond sensitively to the flow of information. This also places the individual in a structural position for the individual to be affected by the actions of others in online interpersonal relationships. As a consequence, the exchange of information during gaming is promoted through the interaction with others, and the level of immersion in online games increases as the individual is able to efficiently establish a gaming network. Such results are considered to contribute to an increase in the degree of IGD. Therefore, the causal relationship between online interpersonal relationships and IGD must be analyzed, and the structural characteristics of online interpersonal relationships must be considered to manage the degree of IGD.

On the contrary, the results of analyzing the relationship between out‐closeness centrality and IGD showed a negative correlation, with higher out‐closeness centrality indicating a decrease in the degree of IGD. Out‐closeness centrality reflects the shortest distance from oneself to another person, and is explained as the direction of the relationship from oneself to the other. In interpersonal relationships, out‐closeness centrality signifies actively working to form and maintain relationships with others, and is described as a characteristic containing sociability. It is also explained as having a characteristic of actively forming and expanding on interpersonal relationships through structural means that make it easier to contact others (Kim et al., 2019). This contributes to forming relationships online, sharing others’ interests, expressing one's feelings, and acting proactively to build a close relationship. The analysis conducted in this study showed that individuals with higher out‐closeness centrality proactively involve themselves in various activities to increase the online communication with the other party rather than participate in the online games, and to be attentive to the interests of others that are revealed online.

Such results are supported by a number of previous studies. Kim et al. (2019) analyzed the centrality of SNS, which showed out‐closeness centrality to have a negative correlation with drinking and smoking. This study reported that individuals who attempted to be close with others had a lower degree of experiencing drinking or smoking, and explained that such tendency to refrain from smoking was linked to the desire to form a close relationship with others. A study by Ennett and Bauman (1996) also suggested that IGD can cause social isolation and be affected by interpersonal relationships. Therefore, university students with a high degree of out‐closeness centrality have a tendency to show interest in various activities with other people to solve such problems and control their gaming to date someone or build a close interpersonal relationship. Leung (2002) also mentioned that the online space for interpersonal relationships has less exposure to a stimulus than the offline space and is an environment in which a person is able to easily express one's feelings and thoughts. When the initiative for interaction increases in such an environment, the individual makes efforts to share various activities with the other person.

A study by Jacobs et al. (2016) also found a negative correlation in the relationship between out‐closeness centrality and smoking. This study explained out‐closeness centrality as the drive to develop close relationships with others and found that it contributes to controlling over smoking by acting as a factor that enables the individual to perform socially positive activities. In addition, such individuals gain correct information on smoking through online communication and consider smoking to be a factor that is socially undesirable and a cause of social isolation. A study by Hall and Valente (2007) similarly reported a decrease in smoking to be associated with a higher degree of out‐closeness centrality. This study depicted such results to have emerged because individuals became attentive to the interests of others to build a positive relationship, and therefore showed control over their interest in smoking, as it could potentially have a negative impact on their ability to build relationships with others. To clarify such contents, future studies must analyze the causal relationship between out‐closeness centrality in online relationships with IGD and consider all the above‐mentioned factors to induce positive interpersonal relationships throughout the management of IGD.

To understand the correlation with online interpersonal relationships, this study reflected the structural characteristics of online interpersonal relationships and conducted SNA on these relationships among university students, with a focus on the degree of social networks, which depicts the interactions that occur between the individuals (Marden, 2005), to analyze the correlation between online interpersonal relationships and IGD. Unlike a number of previous studies of IGD that analyzed the internet game use and satisfaction factors or the negative effects of IGD, this study is significant as it supplemented the limitations of previous studies that were unable to depict the use of internet games with specific characteristics related to the behavior of use (Gyeong et al., 2012).

This study is also significant in that it analyzed the correlation between IGD and online interpersonal relationships as an important predictive factor that induces engagement and interest in IGD, as well as a factor that can induce behavioral changes such as increased levels of immersion, abuse, and maladaptation issues. In addition, this study is considered effective in explaining and understanding the correlation between online relationships and IGD as it used the structural analysis of online interpersonal relationships, which reflects the actual interactions that occur between subjects within a group of college students through SNA analysis and the degree of social networks.

## 5 CONCLUSIONS

The purpose of this study was aimed at analyzing the correlation between online friendship network and IGD. As the results of correlation analysis, the positive correlation between in‐closeness centrality and IGD showed and the negative correlation between out‐closeness centrality and IGD. Online friendship network is considered as a factor contributing to the changes in the level of IGD. Thus, in order to consider changes in the level of IGD, it is necessary to consider the closeness centrality index among the results of SNA which allows structural explanations about the characteristics of the relationship including the relationship direction. However, this study merely analyzed and presented the correlation between the online friendship network and the level of IGD. Therefore, causal analysis should be considered in order to clearly demonstrate these relationships.

## CONFLICT OF INTEREST

The authors declare no conflict of interest.

## AUTHOR CONTRIBUTIONS

All authors were involved in the conception and design of this study, the analysis and interpretation of the data, and the drafting and revision of the article.

### PEER REVIEW

The peer review history for this article is available at https://publons.com/publon/10.1002/brb3.2392.

## Data Availability

The datasets generated and/or analyzed during the current study are from the university students. Thus, after obtaining the subjects’ permission, the datasets are available from the corresponding author on reasonable request.
